# Mental healthcare-seeking behavior of women in Bangladesh: content analysis of a social media platform

**DOI:** 10.1186/s12888-022-04414-z

**Published:** 2022-12-19

**Authors:** Kamrun Nahar Koly, Zarin Tasnim, Sharmin Ahmed, Jobaida Saba, Rudbar Mahmood, Faria Tasnim Farin, Sabrina Choudhury, Mir Nabila Ashraf, M. Tasdik Hasan, Ibidunni Oloniniyi, Rifath Binta Modasser, Daniel D. Reidpath

**Affiliations:** 1 Health System and Population Studies Division, International Centre for Diarrhoeal Disease Research, Bangladesh (icddr,b), Dhaka, Bangladesh; 2 BRAC James P Grant School of Public Health, BRAC University, Dhaka, 1213 Bangladesh; 3 Department of Epidemiology, National Institute of Preventive and Social Medicine (NIPSOM), Dhaka, 1212 Bangladesh; 4 Department of Public Health and Informatics, Jahangirnagar University, Dhaka, 1342 Bangladesh; 5 Department of Public Health, North South University, Dhaka, 1229 Bangladesh; 6 Department of Family Medicine and Public Health Sciences, School of Medicine, Wayne State University, Detroit, MI USA; 7 Action Lab, Department of Human Centred Computing, Faculty of Information Technology, Monash University, Melbourne, Australia; 8 Department of Public Health, State University of Bangladesh, Dhaka, Bangladesh; 9 Department of Primary Care & Mental Health, University of Liverpool, Liverpool, UK; 10 Departmental of Mental Health, Faculty of Clinical Sciences, College of Health Sciences, Obafemi Awolowo University, Ile-Ife, Nigeria; 11 Mental Health Unit, Obafemi Awolowo University Teaching Hospitals Complex, Ile-Ife, Osun Nigeria; 12 School of Public Health, Independent University Bangladesh (IUB), Bashundhara, Dhaka, 1229 Bangladesh; 13 Jeffrey Cheah School of Medicine and Health Sciences, Monash University, Subang Jaya, Malaysia

**Keywords:** Healthcare-seeking behavior, Mental health, Barriers, Women, Bangladesh, Social media

## Abstract

**Background:**

Mental health remains a highly stigmatized area of healthcare, and people often conceal their concerns rather than seek assistance or treatment. The Women Support Initiative Forum (WSIF) is a social media platform established in 2018 to provide expert and peer-led psychosocial support services to women of all ages in Bangladesh. The anonymous nature of the forum means that mental health concerns can be aired without fear of identification.

**Method:**

A content analysis was conducted on the anonymous posts retrieved from the WSIF platform between 8^th^ March 2020 and 7^th^ July 2022. Around 1457 posts were initially selected for analysis which was reduced to 1006 after removing duplicates and non-relevant posts, such as queries about the addresses of the doctors and other non-mental health-related issues. A thematic analysis of the data was conducted using an inductive approach.

**Result:**

The 1006 posts generated four themes and nine sub-themes. All the women mentioned mental health symptoms (*n* = 1006; 100%). Most also mentioned reasons for seeking mental healthcare (*n* = 818; 81.31%), healthcare-seeking behavior (*n* = 667; 66.30%), and barriers to seeking mental healthcare (*n* = 552; 54.87%). The majority of women described symptoms of stress, depression, and anxiety-like symptoms, which were aggregated under common mental health conditions. Mental health symptoms were ascribed to various external influences, including marital relationship, intrafamilial abuse, and insecurities related to the COVID-19 pandemic. A large proportion of posts were related to women seeking information about mental healthcare services and service providers (psychologists or psychiatrists). The analysis found that most women did not obtain mental healthcare services despite their externalized mental health symptoms. The posts identified clear barriers to women accessing mental health services, including low mental health literacy, the stigma associated with mental healthcare-seeking behavior, and the poor availability of mental health care services.

**Conclusion:**

The study revealed that raising mass awareness and designing culturally acceptable evidence-based interventions with multisectoral collaborations are crucial to ensuring better mental healthcare coverage for women in Bangladesh.

**Supplementary Information:**

The online version contains supplementary material available at 10.1186/s12888-022-04414-z.

## Introduction

Mental health issues have been identified as one of the most significant contributors to the global burden of disease and disability [1]. More than 1 billion people have mental illness worldwide. In low- and middle-income countries (LMICs) like Bangladesh, almost 18.7% of adults and 12.6% of children suffer from mental health disorders, particularly depression, anxiety, and stress [2]. Women are more likely than men to suffer from these disorders [3]. This gender disparity is influenced by several biological, hormonal, social, and cultural variables and is more pronounced in LMICs [4–7].

Factors contributing to mental health issues experienced by women are financial dependency, reduced autonomy in decision-making, interpersonal violence, and the social role of women as child-bearers and caregivers [5, 8–11]. In Bangladesh, women are twice as likely to experience common mental health issues (most prevalent mental health conditions such as depression, anxiety etc.) [12]. However, they are only half as likely to access treatment [13].

The main problems affecting men’s and women’s access to treatment are the scarcity of mental health professionals, the mal-distribution of facilities to urban areas, and the improper implementation of national mental health policy [14, 15]. In addition, social and cultural stigma and low mental health illiteracy (low knowledge about mental disorders, prevention of the conditions, management, and referral) have negatively influenced the mental healthcare-seeking behavior among people in Bangladesh [16, 17]. Historically women have lower tendency to seek mental health services worldwide due to different factors [18]. Poverty, low educational status, lack of employment opportunities, low decision-making capacity, inadequate knowledge about mental health conditions, fear of being labeled, perceived impact on marriage, and lower access to healthcare services all impact women’s mental healthcare-seeking propensity worldwide [19–22]. Regarding Bangladeshi women, the national mental health survey reported that only 11.6% sought mental healthcare [23]. However, very few studies reported the pattern of seeking care; therefore, conducting such a study is crucial for devising need-specific interventions for this vulnerable population [13, 21, 23].

The COVID-19 pandemic also had an indirect, adverse impact on mental health inequalities among women [24–26]. The pandemic increased economic uncertainty, reduced employment, and increased women’s exposure to interpersonal violence [27–29].

Understanding the challenges of women with mental health problems is key to improving the nature, quality and reach of services. Unfortunately, for many researchers, policy-makers and mental health professionals, the information available about women’s challenges typically comes from clinical settings (i.e., women already accessing services) or community samples (i.e., cross-sectional surveys or qualitative studies). There is no ready access to women’s spontaneous, every day and personal observations about their mental health challenges.

The Women Support Initiative Forum (WSIF) is an online women-led initiative that offers a wide range of online psychosocial services through it’s website, LinkedIn, and Facebook group and pages [30]. Established in 2018, WSIF was founded by a global mental health researcher and is regulated by seven medical doctors and ten psychologists. There are about 31,000 women who are members of the closed Facebook group (https://www.facebook.com/groups/182300659137448). Eighty percent of members are aged between 20 and 28 years and are distributed across all eight divisions (administrative units) of Bangladesh. WSIF provides online psychotherapy and counseling via expert psychologists and psychiatrists. In addition, it promotes psychosocial wellness and mental healthcare-seeking behavior among women through disseminating mental health-related educational content [31].

The WSIF closed Facebook group also has an “anonymous post” feature in which women can share their challenges anonymously and receive support. The psychologists and psychosocial supporters respond in the comment section of the posts sharing their opinion and guidance in a non-judgemental manner. Other group members who have had similar experiences also respond in the comment section to support to these women. The posters’ identity is anonymous, known only to the group administrators, who maintain strict confidentiality. This feature allows women to express their problems in detail without fearing they will be identified and socially judged.

The anonymous posts provide a unique, unsolicited insight into women’s mental health concerns and treatment-seeking behavior outside a formal healthcare setting. Therefore, our study objective was to explore the pattern of mental healthcare-seeking behavior among women in Bangladesh through social media content analysis of the anonymous posts shared on the WSIF platform. The findings would help to generate evidence on the care-seeking behavior and barriers faced by the women and potentially guide better approaches to service delivery.

## Methodology

### Study design

A thematic content analysis was conducted of the anonymous posts by the Facebook group members of WSIF. Codes were inductively derived from the text data, and themes were defined during analysis. This method paid close attention to the members’ points of view and analyzed their genuine expressions in the posts to better understand the phenomena [32]. We followed the standard reporting protocol using the Standards for Reporting Qualitative Research (SRQR) format and updated the overall manuscript according to the guideline (Supplementary Table 1) [33].

### Study settings and context

Bangladesh has a population of 164 million, and 49.4% are female. According to a Polish-based social media management platform, around 16,868,448 Bangladeshi women actively use Facebook, of which the majority are between 18 and 24 years old [34, 35]. The study was carried out among women in Bangladesh who were members of the WSIF Facebook group and posted anonymously about different social and mental health issues.

### Sample selection and data sources

Two research team members collated the anonymous posts, storing each post as a separate, uniquely identifiable file. The study timeline was from 8^th^ March 2020 to 7^th^ July 2022 since the first case of COVID-19 was identified on 8^th^ March 2020 in Bangladesh [36]. And also by July 2022, the COVID-19 case rate and the morbidity rate decreased significantly and, government-imposed lockdown measures were withdrawn [36]. Moreover, we discontinued selecting new anonymous posts when data saturation was reached. Around 1457 anonymous posts were made within the study period, and following the screening, 1006 of those were retained for analysis. Duplicate posts were removed, as were non-relevant posts seeking information about doctors’ chambers or related to non-mental health issues. All posts were written in Bengali.

### Data analysis

Thematic content analysis was utilized to analyze the anonymous posts [37]. In addition, we also tabulated the frequency distribution of themes and sub-themes. We distributed the 1006 posts among the four team members proficient in Bengali and English, and they familiarized themselves with the posts through iterative reading. Four research team members were medical graduates with Master’s degrees in Public Health and had 1-2 years of training in qualitative data analysis. All were further trained by the lead author, a Global Mental Health researcher with 5-6 years of training in qualitative methodology and multiple publications in international peer-reviewed journals. The four team members individually coded ten anonymous postings to evaluate inter-coder reliability using the Kappa statistic according to Holisti’s Method (1969) [38]. When the score for a particular code was less than 0.6, the team got together to discuss coding issues. The overall final mean was 0.81, showing very strong coding agreement [39]. The analysis team members manually coded the Bengali posts and formalized common data for each code to establish themes and sub-themes. The research team met twice a month for the analytic sessions, where themes and sub-themes were discussed and decided. All researchers summarized the themes and compared the contents. The coding team also identified relevant, illustrative quotes and created a verbatim table in an MS Excel sheet. Team meetings were also held to check each team member’s discrepancies in coding, themes and subthemes and selecting the appropriate quotes supporting the identified themes. The selected quotes were translated into English by the research team members. The anonymous posts were randomly checked by the lead researcher, who was not involved in post-selection and reassessed the analysis.

## Results

**Fig. 1:**
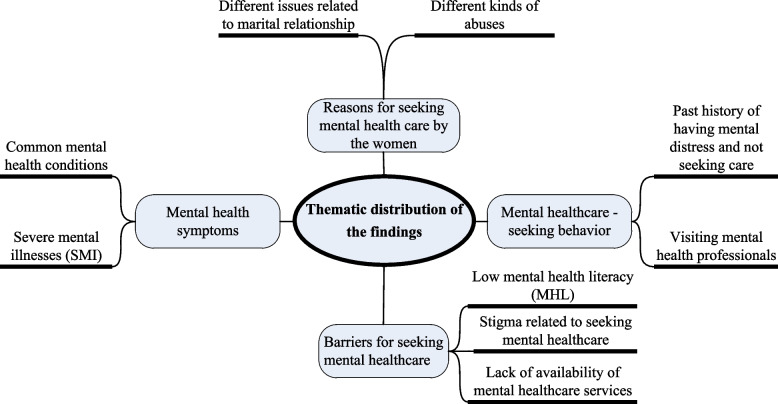
Thematic distribution of the findings

Among the 1006 anonymous posts, 667 (66.30%) were about mental healthcare-seeking behavior, and 552 (54.87%) were related to barriers to seeking mental health support. The findings were summarized in four themes and nine sub-themes in Table 1 and illustrated in Fig. 1. Apart from the mentioned quotes in the result section, the others are given in supplementary section (Supplementary Table 2).

**Table 1:** Thematic distribution of the findings

Theme	Sub-themes	Frequency (Percentage)
1. Mental health symptoms	1.1. Common mental health conditions	911 (90.56)
	1.2. Severe mental illnesses (SMI)	95 (9.44)
2. Reasons for seeking mental health care by the women	2.1. Different issues related to marital relationship	396 (48.41)
	2.2. Different kinds of abuses	422 (51.59)
3. Mental healthcare -seeking behavior	3.1. Past history of having mental distress and not seeking care	552 (82.76)
	3.2. Visiting mental health professionals	115 (17.24)
4. Barriers for seeking mental healthcare	4.1. Low mental health literacy (MHL)	426 (77.17)
	4.2. Stigma related to seeking mental healthcare	120 (21.74)
	4.3. Lack of availability of mental healthcare services	6 (1.09)

### Theme 1: mental health symptoms

The anonymous posts (*n* = 1006; 100%) described mental health symptoms, which could be summarized into two major sub-themes: (i) common mental health conditions (*n* = 911; 90.56%) and (ii) severe mental illnesses (*n* = 95; 9.44%). The posts in which women expressed low mood, hopelessness, lack of concentration, fear, worry, distress were categorized under common mental health conditions as per the evidence [40–44]. On the other hand, those posts which expressed suicidal thoughts or attempts of suicide were classified as severe mental illnesses [45, 46].

#### Common mental health conditions

The majority of the posts reported depressive symptoms followed by symptoms of stress and anxiety, reporting various stressful life events affecting their day-to-day activities. Women experiencing these mental health issues also described worries, insecurities, frustration, low self-esteem, and hopelessness.

*I used to dream of being a vocal artist and news presenter. But I could not pursue my career after the birth of my first child. When I was preparing again to return [to my career], I conceived for the second time. Now, I am frustrated by my stalled career and getting more hopeless day by day.*

- Anonymous post 425

The ongoing pandemic was observed to have a direct effect on the mental health of some women. The women commonly shared fear of infection and tension after being infected with COVID-19. Women also reported different issues related to the pandemic, such as job insecurity and restriction of social activities. One of the posts illustrated the magnitude of the impact.

*This COVID-19 situation has turned my life upside down. I lost my father (because of the infection). Now I am continuously afraid of losing other family members too. My husband does not let me visit anyone, even my siblings. I also lost my job during the early pandemic and could not sleep well. Sometimes I have terrible nightmares.*

*-* Anonymous post 144

#### Severe mental illnesses

In addition, some women’s posts indicated severe mental illness, including suicidal thoughts and suicidal attempts. They reported traumatic events as the triggers, such as the death of people close to them or the experience of constant abusive behavior by extended family members.

*My mother passed away last year, and I have been trying to cope with this trauma ever since. I cannot concentrate on my daily activities, and no one understands how I feel. This pain seems unbearable to me. I am not even able to help myself. I am constantly thinking about suicide. I feel helpless.*

- Anonymous post 392

### Theme 2: reasons for seeking mental health care by the women

The majority of the posts (*n* = 818; 81.31%) revealed that women sought mental healthcare when dealing with various issues related to marital relationships (*n* = 396; 48.41%) and different kinds of abuse (*n* = 422; 51.59%) they endured.

#### Different issues related to marital relationship

Marital relationship issues were documented as one of the major causes of distress and seeking mental healthcare. They were stressed by the low levels of emotional support they received, including inadequate attention, poor communication, lack of commitment, and absence of mutual understanding by their husbands. A lack of financial support from their husbands, particularly as it related to their own or their children’s health and education, also had a negative impact on their mental health. One woman shared her frustrations thus:

*My husband is extremely busy with his mobile phone, friends, and socializing. He is not emotionally connected with my daughter or me anymore. He gets irritated while spending time with us. He is not even concerned about our financial needs and does not bear any expenses for our daughter. I am very worried and frustrated with his behavior.*

- Anonymous post 190

Other women revealed low marital physical intimacy, abnormal sexual fantasies, pornography addiction and infidelity by their male partners as some of the primary reasons for their distress. One post stated:

*I accidentally opened my husband’s mailbox and saw an improper conversation with another woman. I feel cheated and deceived. I am continuously worried about him having sexual relationships with other women, which negatively affects my daily life.*

- Anonymous post 67

In addition, some women expressed stress influenced by the actions of their parents-in-law. Women often felt judged by the parents-in-law for their physical appearance and their parent’s financial status. Some women also reported an unsupportive attitude of in laws regarding career choices or participation in household chores that contributed to marital disharmony. Interference by the parents-in-law in family planning decisions also affected the posters mental health.

*I was expected to manage all the household chores at my parents-in-law’s house while managing a full-time job. After repeated conflicts with them, my husband and I had to get a separate place to live. Even after I became pregnant and gave birth to my child, my parents-in-law did not visit me, and I did not receive any support from them.*

- Anonymous post 83

#### Different kinds of abuses

In more than half the posts, abuse experienced in childhood, which resurfaced in later adult life, was the reason for seeking peer support. The abuse was mainly attributed to parents, husbands, parents-in-law, or other closely related family members. Women presented with distress from enduring such abuse and one of them stated:

*I have been dealing with my toxic parents since childhood. My mother used to beat me inhumanly for a simple mistake and often locked me up at home. My body still has the scars of my past bruises and abrasion because of her physical torture. I always used to be discouraged verbally. As a result, I am suffering from overthinking and constant sadness. I have lost my confidence, which badly affects my career growth and relationship.*

*-* Anonymous post 78

In most cases, women were found to be suffering from emotional abuse, which came in the form of controlling behavior, negligence, and disrespect and was sometimes compounded with verbal abuse. The verbal abuse was illustrated through bullying, mocking, and teasing by the family members and relatives. Such behavior was commonly male partners due to the influence of the parents-in-law, and one of them mentioned:

*I was a victim of bullying and physical torture by my parents-in-law and husband. I constantly heard negative comments regarding not having a baby, low salary and skin color. My husband is manipulated by my parents-in-law, although we had a love marriage. I cannot tolerate it anymore. I am breaking down day by day.*

- Anonymous post 174

A few women disclosed that they experienced sexual abuse including improper touching and sexual assault by outsiders. The experience had a longer-term impact on their lives and affected their well-being.

*I was sexually assaulted several times by different people, at different ages of my life. One of the abusers repeatedly touched my genitals and lower abdomen, saying, “you are my buina (sister)”. I cannot forget those incidents no matter how much I try.*

- Anonymous post 38

### Theme 3: mental healthcare-seeking behavior

A majority of posts (*n* = 667; 66.30%) described the pattern of mental healthcare-seeking of the women. The coded posts were summarized into two sub-themes (i) past history of seeking mental health care (*n* = 552; 82.76%) and (ii) visiting mental healthcare professionals (*n* = 115; 17.24%).

#### Past history of having mental distress and not seeking care

The majority of the posts described mental health challenges in the past, indicating that the posters did not receive any professional psychosocial support despite their need.

*My husband lives abroad. In our 21 years of marriage, he mentally tortures me every time he visits Bangladesh. Furthermore, my spouse has cheated on me several times, which I can no longer tolerate, but nobody at my parents-in-law understands my issue. I have been going through major distress for a long time but I did not seek mental health care because it is very expensive. I take sleeping pills whenever I feel overwhelmed. I don’t know what to do.*

- Anonymous post 482

One of the women quoted-

*Three years before, I had a terrible miscarriage in the ninth week of my pregnancy, and ever since, I hallucinated a baby who is calling me a mother. I could not make anyone understand how unbearable this feeling was! It got worse gradually however I did not know where to get help as no one in my family related to my issue. These days, I am only counting the days to meet my child.*

- Anonymous post 320

#### Visiting mental health professionals

Relatively few posts mentioned visiting a mental health professional, mostly psychiatrists. More than half of those who did seek professional services, however treatment compliance was not clearly reported. Some of the women posted about the reasons for their non-adherence to mental health support.

*Last year, I visited a psychiatrist. In the beginning, I was diagnosed with clinical depression but later I got to know I have borderline personality disorder. But I had to stop taking treatment as my family was unsupportive and denied my mental health issues.*

- Anonymous post 245

Some women also expressed their interest in obtaining professional help in future.

*I feel very stressed and worried about my marital life. I have been struggling very hard to keep a stable relationship with my husband for a long time. I need suggestions regarding an expert psychologist for couples counseling. It would also be beneficial to have information about online consultations.*

- Anonymous post 490

### Theme 4: barriers to seeking mental healthcare

The majority of the anonymous posts (*n* = 552; 54.87%) directly and indirectly reflected on different barriers to seeking mental healthcare. Analyzing these posts, three major sub-themes emerged as: (i) low mental health literacy (*n* = 426; 77.17%) (ii) stigma related to seeking mental healthcare (*n* = 120; 21.74%), and (iii) lack of availability of mental healthcare services (*n* = 6; 1.09%) (Table 1).

#### Low mental health literacy

A large share of posts mentioned inadequate mental health literacy as one of the major challenges for women in seeking care. For example, one woman posted the following.

*I went through long-term emotional distress because of toxic and failed relationships. I try to keep myself busy with work, but it is difficult to forget the trauma. Is this a phase that will pass, or is this a mental illness? How can I overcome this trauma?*

*-* Anonymous post 43

Some also mentioned a low understanding of the types of mental health services providers. A few did not know where to get professional psychosocial support.

*I have been suffering from endless anxiety after getting divorced from my husband and hoping it will end soon. I hesitated to seek professional help because I constantly fear being labelled as vulnerable and losing custody of my child. I live abroad and am currently visiting Bangladesh, so I have no idea where and how to seek help.*

- Anonymous post 509

#### Stigma related to seeking mental healthcare

Concern about social stigma was identified as a barrier to seeking mental healthcare. Hiding the psychological symptoms out of fear of societal judgment was also mentioned in a few posts. Some women also told of how family members encouraged them not to reveal their emotional issues to any outsiders. One woman posted:

*My family was never supportive of my mental health condition. They used to lock me up in a room so that I couldn’t share my issues with anyone. Now I realize that if I could get counseling or any other timely support, maybe I could get rid of this trauma.*

- Anonymous post 46

Another post recounted:

*I have my parents, siblings, husband, and a child. But I cannot share my feelings with anyone because they are all judgmental. They don’t consider my mental health important. Would you please suggest ways to control stress?”*

- Anonymous post 9

Some posters also mentioned no improvement in their mental health in spite of receiving care. They felt judged by the mental health professionals, and they stopped the treatment. One woman posted,

*I am very keen to learn psychology, so I study online articles to learn. This [new understanding] makes me feel as if my psychologist was only offering me the most basic suggestions. Also, I felt that she was so disappointed in me. I expected her to guide me by exploring my internal struggles. But she had strong opinions and was attempting to make decisions for my life. As a result of her actions, I decided to quit taking sessions.*

- Anonymous post- 133

Another woman wrote,

*My parents always try to influence every decision in my life. Unfortunately, when I visited a psychiatrist, he repeated the same statements as my parents and prescribed some antidepressants. I could not share my problems with him in detail, and subsequently, I stopped receiving treatment*.

- Anonymous post 361

#### Lack of availability of mental health care services

The absence of psychiatrists within reach was mentioned in a few of the posts. Some of the posts also identified a lack of affordability and distance as issues.

*I have been dealing with mental health issues for a long time, but we don’t have any available psychiatrists in our area. So, my need for professional mental health care remains unmet.*

- Anonymous post 303

*I have been suffering from anxiety and overthinking for a long time. But I cannot receive counseling services immediately due to our financial condition. I am currently staying in my village, so I can’t travel to Dhaka to use the counseling services there.*

- Anonymous post 329

## Discussion

Globally the mental health burden in women is increasing. However, particularly in LMICs, there are limited studies to investigate gender-specific factors [47–51]. Our study identified social and cultural aspects associated with women’s personal experiences of mental health issues. The data, importantly, were also drawn from spontaneous, every day and private observations about their mental health challenges. This makes an important contribution beyond facility-based studies or formal, sampled research. Using a social media platform, our study adopted a unique qualitative approach to identify needs, help-seeking behavior and barriers for receiving mental health care among Bangladeshi women.

According to our findings, most women reported the symptoms of depression, anxiety, stress with, in extreme cases, suicidal ideation and suicidal behavior. Prior studies have also reported these mental health burden among women in Bangladesh [9, 23]. In keeping with other research, our study also reflected pandemic-related issues such as fear of infection, restriction of social activities, and job insecurity that negatively affected women’s psychological well-being [52–54]. The issues the women raised in their posts may also point to the reasons for their higher vulnerability to mental health issues. Specifically, in similar country settings, their burden might be influenced by gender-specific roles, socio-economic factors, and family structures [55–58]. It would be vital, in these circumstances, to train professionals to provide psychosocial support to women to address these unique challenges, especially during public health emergencies such as the COVID-19 pandemic [59–61].

Additionally, our study identified unhealthy marital relationships as one of the most common reasons for women to seek mental health support on social media platforms. Most married women who posted, reported dealing with marital conflicts compounded by a perceived low emotional attachment to their husbands, unsupportive parents-in-law, addiction to pornography, and infidelity. Studies from neighboring countries also reported such issues causing a higher likelihood of mental health conditions, low self-esteem, functional impairment, and even suicidal ideation among women [62–66]. The community-based promotion of premarital therapies, family therapies, women’s rights in marital relationships, and access to couple counseling services could be important initiatives to increase awareness about marital intimacy and harmony [67, 68].

Women also described challenges like abusive behavior and violence perpetrated by husbands and parents-in-law in their lifetime, which was also exacerbated by the COVID-19 pandemic [69–72]. Like other developing countries, the cultural tendency to live in extended families causes socio-economic dependency, and the predefined social expectation of women increase the possibility of violence, which impacts their mental health [11, 69, 70, 73]. Other studies have also highlighted the negative impact of parents-in-law on women’s autonomy and their decisions about career, child-rearing, and family planning [69, 70, 74, 75]. The health and well-being of women has been found to be affected by conflict with the husbands’ family, intimate partner violence (IPV), lack of autonomy, and violation of sexual and reproductive health rights (SRHR) [11, 61, 75–78]. Therefore, it is instrumental to increase awareness about women’s constitutional and SRHR rights and ensuring the availability of 24/7 telecommunication services to prevent violence against women in Bangladesh.

Women in our study highlighted various early life adversities, especially by parents and sexual abuses by close networks. South Asian parents have influence over their child’s decision-making and social activity, which increases the likelihood of childhood maltreatment that has a long-lasting impact on their mental health [79, 80]. Evidence supports that the abusive behavior of parents is perceived as permissible in such settings to assure children’s disciplined behavior and better life outcomes [79, 80]. Ongoing psychological distress following emotional and sexual abuse at an early age is well-identified in the literature [79]. Advocacy of evidence-based healthy parenting practice and age-specific training in sex education should be promoted.

Our study also identified the lack of mental healthcare-seeking among women in Bangladesh despite their experience of various psychological challenges, traumatic events, and early life adversities. Low mental health literacy, social stigma, and the poor availability of services were reported factors that reduced mental healthcare-seeking behavior. It is highlighting the convenience of seeking anonymous peer help on social media-based platforms such as WSIF. A similar observation was made about Chinese women who found it more convenient to talk to family members, social networks, or even use online resources rather than visiting mental health professionals [81]. Among WSIF members, seeking peer mental health support on-line could be attributed to the ease of access, low cost, and anonymity, as well as the opportunity to interact with others who have shared similar experience [82, 83]. This can provide a sense of companionship and self-management.

Our study highlighted the different barriers to seeking mental health services among women, such as limited ideas about symptoms and care pathways, fear of judgment, and lack of familial support. Moreover, experiencing IPV and women’s economic dependency might also affect their care-seeking behavior in our study, which also corroborates with past literature [61]. Besides only a few women reported visiting professionals but with a disrupted therapeutic continuation due to low mental health literacy and a lack of understanding of psychosomatic symptoms [17, 84, 85]. Furthermore, our study found that women had limited access to quality mental health services, which is a significant factor in the higher mental health burden and treatment gap among women in Bangladesh [84, 86]. This lack of professional mental health services in other developing countries are also caused by low mental health literacy, stigmatized attitudes, low affordability, and a workforce shortage [85, 87, 88].

Therefore, the observed poor mental healthcare-seeking pattern of women strongly supports the barriers mentioned above to cause sub-optimal or delayed use of mental health services which impedes the recovery process [15, 89]. Hence, it is important to address policy-level attention to promoting psychosocial support services through behavioral change communication strategies. Furthermore, it is crucial to integrate mental health in primary healthcare services, adopt a task-shifting approach, and provide alternative digital mental health interventions to influence the mental healthcare-seeking behavior of women in Bangladesh [90]. Moreover, a few women expressed interest in seeking professional care, which could be due to the increased availability of digital-based mental health-related information during the pandemic. Thereby, the structured use of social media and digital services for disseminating appropriate information and quality mental healthcare should be a prime concern at the government level, especially during such a crisis of ongoing pandemic.

### Strengths and limitations

This study has several limitations. The data were collected from postings to a social media platform. The postings may be incomplete, selective, or untruthful. Some posts may represent the immediate reaction to a current event, the perspective of the person sharing the post, or and actual or long-term scenario. It is not always possible to disentangle this from the posts. It was not possible to obtain detailed information from the family members, including the women who shared the posts, to understand the contextual factors contributing to their mental health challenges or care-seeking behavior. Most posts did not possess adequate information regarding expert diagnosis of the mental illness or clinical prognosis. Moreover, the sociodemographic information about the women was missing due to anonymity. Finally, the women who are members of the forum, and the subset of those women who post about mental health issues, are not representative of the population. They are, for instance, likely to be educationally and socioeconomically distinct.

Nonetheless, the opportunity to “peek into” the unsolicited and spontaneous mental health issues of women in an LMIC setting is rare and provides rich insights that may be missed in facility-based studies or research studies formally sampling women. This study is the first of its kind in Bangladesh to explore the mental healthcare-seeking behavior of women from their own experiences, trauma, struggles, and perspectives. The study identifies vital information about mental health issues, possible reasons for seeking care, and barriers to receiving mental health care among women in Bangladesh. It would assist the relevant stakeholders take initiatives for effective community engagement and mobilization, which would support women using social media.

## Conclusion

This study is a critical snapshot depicting the scenario of mental health issues and services from the perspectives of a group of females, especially when a pandemic is continuing. During the pandemic, there has been a quick transition to delivering mental health care based on digital platforms. Hence it is essential to learn from individuals at the forefront of such innovations. Our research of anonymous posts on social media revealed a range of immediate reactions and experiences to these changes. However, to inform future planning and implementation of the policy, larger scale studies, both qualitative and quantitative, are required to investigate the situation. Moreover, findings of the study echoed the need for evidence-based and culture-specific strategies for women’s empowerment in Bangladesh particularly for mental health needs. A multidisciplinary approach is necessary to endorse feasible and cost-effective interventions to increase the accessibility of the mental health care service to women especially during an emergency like COVID-19 pandemic. In addition, the involvement of the different media and digital platforms would be crucial to raising mass awareness and eliminating the stigma to influence the overall mental healthcare-seeking behavior in the community.

## Supplementary Information

**Additional file 1: Supplementary Table 1.** Standards for Reporting Qualitative Research (SRQR). **Supplementary Table 2.** Table of Quotes.

## Data Availability

The datasets utilized for the current study are available upon reasonable request to the corresponding author.

## Abbreviations

COVID-19: Corona Virus Disease- 19
LMIC: Low- and Middle-Income Country
HIC: High Income Country
IPV: Intimate Partner Violence
SRHR: Sexual and Reproductive Health Rights
WHO: World Health Organization
WSIF: Women Support Initiative Forum
