# Supplementary material for: Mental healthcare-seeking behavior of women in Bangladesh: content analysis of a social media platform
Source: BMC Psychiatry. 2022 Dec 19;22:797. doi: 10.1186/s12888-022-04414-z (PMC9760542; doi:10.1186/s12888-022-04414-z)
Supplement: Supplementary file 1 — Additional file 1: Supplementary Table 1. Standards for Reporting Qualitative Research (SRQR). Supplementary Table 2. Table of Quotes. [file 12888_2022_4414_MOESM1_ESM.docx]

**Supplementary Table 1: Standards for Reporting Qualitative Research (SRQR)**

| **No.** | **Item** | **Description** | **Page no** |
| --- | --- | --- | --- |
| **Title and abstract** | | |  |
| 1 | Title | The title indicates the qualitative approach of the study (content analysis) | Page 1 |
| 2 | Abstract | The abstract is formatted in accordance with the requirements of the journal. | Page 2 |
| **Introduction and aim** | | |  |
| 3 | Problem formulation | A description of the phenomenon under investigation and a review of relevant theory and empirical work are provided. | Page 4-5 |
| 4 | Purpose or research question | The purpose of this study has been stated in the aim | Page 5 |
| **Methods** | | |  |
| 5 | Qualitative approach and research paradigm | The empirical data is obtained from the anonymous posts published in Facebook based online platform Women Support Initiative Forum (WSIF) of Bangladesh and analyzed using Thematic Content Analysis | Page 5-6 |
| 6 | Researcher characteristics and reflexivity | The lead researcher is trained in Global Mental Health Research. The remaining researchers have a background in public health and healthcare sciences. The authors’ contribution is presented under the heading “Authors Contribution”. | Page 1, 6, 15 |
| 7 | Context | The setting for the context of the study is presented in introduction and methodology | Page 4-6 |
| 8 | Sampling strategy | The Facebook group members of WSIF who posted anonymously for seeking assistance with mental health issues during the study period. | Page 6 |
| 9 | Ethical issues pertaining to human subjects | The study was approved by the Ethics Review Committee of Uttara Adhunik Medical College, Dhaka, Bangladesh. | Page 16 |
| 10 | Data collection methods | The data source, study timeline, iterative process, etc were described. | Page 5-6 |
| 11 | Data collection instruments and technologies | The study was a content analysis therefore there was no need of interview guidelines and recording devices. | N/A |
| 12 | Units of study | The total number of anonymous posts were 1457 | Page 6 |
| 13 | Data processing | The coding process was described in | Page 6 |
| 14 | Data analysis | Thematic content analysis method was used to analyze the anonymous posts and distribution of themes and sub-themes were represented in Table 2 and Figure 1 | Page 6, Table 2 and Figure 1 (Supplementary File 1.B and 1.C) |
| 15 | Techniques to enhance trustworthiness | We have evaluated the inter-coder reliability and reassessed the analysis | Page 6 |
| **Results/findings** | | |  |
| 16 | Synthesis and interpretation | The result was synthesized into four major themes and nine sub-themes | Page 7-12, Table 2 and Figure 1 (Supplementary File 1.B and 1.C) |
| 17 | Links to empirical data | Several quotes substantiate the analytic findings | Page 7-12  Table 3 (Supplementary File 1.D) |
| **Discussion** | | |  |
| 18 | Integration with prior work, implications, transferability, and contribution(s) to the field | The discussion section began with a brief description of the key findings, followed by a comparison to previous studies and potential implications. | Page 12-14 |
| 19 | Limitations | The limitations of this study were clearly stated. | Page 14-15 |
| **Other** | | |  |
| 20 | Conflicts of interest | The authors report no conflicts of interest | Page 16 |
| 21 | Funding | This study received no grant funds | Page 16 |

**Reference**

O’Brien, B.C., Harris, I.B., Beckman, T.J., Reed, D.A., & Cook, D.A. (2014). Standards for Reporting Qualitative Research: A Synthesis of Recommendations. *Academic Medicine, 89*(9), 1245-1251. <https://doi.org/10.1097/ACM.0000000000000388>

**Supplementary Table 2: Table of Quotes**

| **Theme** | **Subtheme** | **Verbatim** |
| --- | --- | --- |
| 1. Mental health symptoms | - 1. Common mental health conditions | - *“I got divorced with my husband 2 years ago. I have been through severe emotional distress since then. Every night when try to sleep, I keep overthinking on the reasons of my marriage not working, my ex-husband cheating on me, my child being fatherless for 2 years, and not loving myself enough etc. All these questions burn my heart every day. I am exhausted and feel insecure all the time”* (Anonymous post 8). - *“I had 3 years gap after completion of my graduation. My friends and juniors are academically successful in their career. But I am severely frustrated and depressed with my career. Even my post-graduation is being delayed due to the ongoing pandemic. I cannot concentrate on my study properly. I have lost my confidence”* (Anonymous post 68). |
|  | - 1. Severe mental illness (SMI) | - *“I have experienced lots of adverse situations in life. Neglect in joint family, sexual abuse in childhood, failed relationships, mental torture by in laws, lack of support from husband, and post-partum depression all these incidents have ruined my happiness. I am having severe mental breakdown. I feel like committing suicide with my children”* (Anonymous post 99). - *“My parents are very toxic. They always claim themselves to be the best parents and keep telling what they did for me. They force me to accept all of their decisions in my life. They are making my life miserable and not ready to listen to me. I am being suicidal for them but they pretend to be the one who are suffering”* (Anonymous post 119). |
| 1. Reasons for poor mental health | - 1. Different issues related to marital relationship | - *“Though he (Husband) has a very good job now, he spends very little on me. He is reluctant to spend on my study and health. Even he neglects me when I ask him to go for the doctor’s appointment with me. He says, ‘why don’t you do it by yourself!’ I cannot accept it and feel very insecure”* (Anonymous post 89). - *“Our conjugal life was not normal. I was never comfortable in physical intimacy with my husband because of his abnormal sexual activity. He used to watch pornography on a regular basis. Even if I disagree to be intimate with him, he used to complain to his parents about this issue! Deep inside, I am really unhappy and severely stressed”* (Anonymous post 93). |
|  | - 1. Different kinds of abuses | - *“My father managed to transfer my boyfriend to another branch out of Dhaka according to a pre-plan with my boss. He wants me to marry someone selected by him. I was confined in the house by my father and was not allowed to go to the office. I am helpless and hopeless at this moment”* (Anonymous post 67). - *“My mother-in-law is extremely dominating. She tries to control and manipulate my husband including me in every possible way. She also demands all the money earned by my husband. My husband does not agree to stay separate from my in-laws. I am extremely irritated with her dominating and controlling attitude which is affecting my mental health.”* (Anonymous post 486) - *“My father sexually assaulted me multiple times since I was a 9 years old child. He tried to convince me that those incidents were normal. My mother was always silent on this issue. She was afraid of my father. Whenever I close my eyes, those terrible memories keep coming. I hate my body. I cannot sleep at night even with sedatives. I just want my pain to be lessened”* (Anonymous post 327). |
| 1. Mental health care seeking pattern | - 1. Past history of having mental distress and not seeking care | - *“In last few years, I feel the presence of another person within myself. I cannot differentiate between the reality and my imaginations. Sometimes I even hallucinate and feel being another entity. This situation is gradually getting worse. I cannot seek professional help as I will not be able to explain my problems to the mental health professional being an entirely different entity other than myself.”* (Anonymous post 484) - *“I have been through repeated failures in my academic life. Consequently, I am suffering from low self-confidence. I am struggling very hard to concentrate on the study for the preparation of government job. I have tried every possible way to help myself. I cannot go for counselling right at this moment as I just got married and do not know how my family members would accept this issue.”* (Anonymous post 439) |
|  | - 1. Visited mental health professionals | - *“I have been suffering from clinical depression and receiving medications and counselling for last few years. But unfortunately, I could not continue receiving counselling because of my loneliness. I could not cope with my depression as my family is not supportive in this regard. They perceived my sickness as my laziness. Doctors and therapists have their limitations. They cannot always stay with me. Even if I fall down, I have no one to hold me back”* (Anonymous post 341). - *“My parents always try to influence every decision in my life. My opinions have never been valued by them. Unfortunately, my psychiatrist just echoed the statements of my parents while he was counselling me and prescribed me some antidepressants. I thought I would be able to share my problems with him. Subsequently, I stopped receiving counselling*.” (Anonymous post 361) - *“I cannot control my anger even after trying a lot. I need suggestions regarding an expert non-judgmental psychologist to manage anger issue, who counsels and listens to the patient with adequate time including patient’s family members if required”* (Anonymous post 441) - *“I strongly believe my husband is a patient of narcissistic personality disorder and I am being narcissistic abused. Can anyone recommend me any psychiatrist or counselor who is expert in this?”* (Anonymous post 172). |
| 1. Barriers for care-seeking | - 1. Low mental health literacy (MHL) | - *“How long depression or anxiety can persist after pregnancy? I have lost my child on May, 2021 but I cannot be normal even in this month of August. I feel palpitation, nausea and sick despite of all reports being normal. Additionally, I am suffering from fear of being affected by corona also. I'm irritated and upset all the time and feeling completely helpless. I am not sure about whether these issues will resolve spontaneously or I should get professional help to recover from the past trauma.”* (Anonymous post 41). - *“We have passed 4years of our married life. We love each other a lot but very unfortunately, despite our constant effort, we could not be physically intimate to each other till now. Sometimes I try to convince myself saying that there is no such thing as physical attachment. Sometimes I feel worthless. Is this a psychological problem? I do not know what I should do”* (Anonymous post 349). |
|  | - 1. Stigma related to seeking mental healthcare | - *“I have been a patient of depression for many long years. Sometimes when there are panic attacks, he (husband) helps me a lot. But other than physical illness, depression never matters”* (Anonymous post 282). - *“My family was never supportive while I was struggling with my emotional trauma from a toxic relationship. Until a particular time, they locked me up in a room. Besides, I had to financially depend on my aunt. Now I realize if I could get counseling or required support at the proper time, maybe I could get rid of this trauma.”* (Anonymous post 46). - *“I have been suffering from ovarian cancer for past few years. During the whole period of my sickness, I have been through severe mental breakdown due to my physical complications at this very early age of 27. My parents strictly said not to share the information in public, but I needed the support for my mental health, so I posted here to get some advice”* (Anonymous post 297). - *“I have been suffering from depression since long ago. But I could not share this issue with my family members and seek professional help as it’s a taboo in our society. Seeking help for my mental health issues has been delayed due to this hesitation which is escalating the problem day by day”* (Anonymous post 437) |
|  | - 1. Lack of availability of mental healthcare services | - *“I am exhausted with my partner’s irresponsible attitude towards me. I cannot leave him as I am both emotionally and physically extremely attached with him. My parents are not supportive as well. I often have panic attacks while being in constant worry for these issues. Unfortunately, no mental health professional is available nearby my location at this moment. My mental health condition is gradually getting worse”* (Anonymous post 303) - *“I am a patient of both bipolar disorder and borderline personality disorder. I used to receive both medication and counselling for my mental health conditions. But unfortunately, a few months back, my psyciatrist suddenly died for his health issues. I could not find an alternative of him to continue my treatment”* (Anonymous post 468) |
